# Alanine-fortified tomatoes relieve the acute alcohol-induced adverse effects in healthy men: a randomized cross-over study

**DOI:** 10.1186/s12986-016-0087-9

**Published:** 2016-04-05

**Authors:** Shunji Oshima, Sachie Shiiya, Yoshimi Tokumaru, Tomomasa Kanda

**Affiliations:** Research & Development Laboratories for Innovation, Asahi Group Holdings, Ltd., 1-21, Midori 1-chome, Moriya-shi, Ibaraki 302-0106 Japan

**Keywords:** Alanine-fortified tomato, Alcohol concentration, Urinary pH, Sedative sensation

## Abstract

**Background:**

Little is known about the effects of dietary components on the regulation of the gastric emptying rate of alcohol and its impact on alcohol metabolism. We recently found that the crude water-insoluble dietary fibers from several types of botanical foods maintained aqueous ethanol solutions. Additionally, the ethanol-absorbing ability of the dietary fibers correlated with the inhibition of the blood ethanol elevation by delaying gastric emptying. Moreover, we found that the synergism between tomatoes and alanine to reduce the absorption of alcohol in rats was attributable to the effect of alanine on precipitates, such as the crude water-insoluble dietary fibers of tomatoes. In the present study, we assess whether an alanine-fortified tomato (AFT) is effective in relieving acute alcohol-induced adverse effects by lowering the alcohol action in healthy human volunteers following the ingestion of alcohol with a meal.

**Methods:**

Twenty healthy males ingested the AFT or sugar as the control, with 1.2 g/kg of alcohol and a micronutrient-fortified meal in a randomized cross-over study. Breath alcohol concentrations were temporally measured, and blood and urine samples were obtained during the trial. The study protocol was repeated with the AFT and sugar groups reversed 4 weeks later.

**Results:**

Various analyses were performed using the data from the 15 subjects. The breath alcohol concentrations significantly decreased when AFT was ingested. A decrease in the urinary pH was also noted following the ingestion of AFT. Moreover, the sum of seven sedative scores as subjective sensation after alcohol ingestion was significantly reduced by AFT the next morning.

**Conclusions:**

Our study demonstrates that the simultaneous ingestion of AFT under the consumption of excess alcohol and a micronutrient-fortified meal relieved the acute alcohol-induced adverse effects in male volunteers. These results are consistent with the effectiveness observed in rats as previously reported.

## Background

Food consumption prior to drinking alcohol decreases blood alcohol concentrations (BAC) and increases the level of alcohol eliminations, as is commonly advised: “Don’t drink alcohol on an empty stomach.” For example, the oral intake of sucrose or fructose has been shown to stimulate the elimination of alcohol from the bloodstreams of healthy subjects or alcoholics [1–3]. Alanine, is an amino acid that likely contributes to the formation of pyruvate by oxidative deamination. The metabolic pathways of alanine could generate nicotine adenine dinucleotide (NAD+), which facilitates alcohol oxidation in the liver via the conversion of pyruvate to lactate [4, 5]. Ramchandani et al. reported that intravenous alcohol administration after eating meal increased alcohol elimination rates that was not due to the specific interaction with meal constituents involving high levels of protein, fat, or carbohydrates [6].

However, we believe that food plays a more intensive role in alcohol pharmacokinetics, specifically to delay the gastric emptying of alcohol as a major factor involved in alcohol absorption. Several previous studies have shown that the delayed gastric emptying of ethanol strongly decreases BAC [7–12]. However, little is known about the effects of dietary components on the regulation of the gastric emptying rate of alcohol and its effect on alcohol metabolism. Jones et al. reported that alcohol ingestion after eating a meal decreased BAC, regardless of the specific interactions with meal constituents [13]. Therefore, it is likely that the most important role of food in the context of alcohol ingestion is to prolong the retention of alcohol in the stomach regardless of the nutrient(s) involved.

Recently, we have demonstrated that the crude water-insoluble dietary fibers of several types of botanical foods besides tomatoes absorb ethanol-containing solutions [14]. In addition, the absorption of ethanol correlated with the inhibition of the blood ethanol elevation by delaying gastric emptying. Subsequently, we identified alanine as a dietary nutrient that can synergize with crude water-insoluble dietary fibers of tomatoes to lower the BAC in rats [15]. Furthermore, the administration of tomato juice supplemented with alanine ameliorated the spontaneous motor activity in rats following the administration of a high dose of ethanol. We concluded that the precipitate of tomato and alanine synergized to decrease ethanol absorption.

Next, preliminary human studies were conducted to access the BAC lowering effect of the tomato and alanine combination. Tomato juice containing 0.6 g of water-insoluble dietary fiber with 5 g of alanine decreased the BAC and relieved the subjective sensations of drunkenness when moderate alcohol (500 mL beer) was ingested under fasting condition (unpublished data). Although tomatoes combined with alanine could have potential clinical significance regarding the adverse effects of alcohol ingestion, the verification of its effectiveness is needed in further studies. This is particularly essential as it has been established that the ingestion of a meal prior to the consumption of alcohol reduces the BAC and increases the level of alcohol elimination, as described above. However, few human trials have focused on the effectiveness of certain agent(s) or foods on BAC elevation following alcohol ingestion after or during meal consumption regardless of the type of food. Therefore, we conducted a randomized cross-over study to evaluate whether an alanine-fortified tomato (AFT) can modify the metabolism of alcohol. In addition, we assessed if AFT could also relieve the acute alcohol-induced adverse effects under the conditions of alcohol ingestion with a meal in human volunteers.

## Methods

### Subjects

This study was proposed and approved by the Ethics Committee of Huma R & D Co. Ltd. Informed consent was obtained from 78 healthy Japanese men older than 20 years who participated in baseline screening. Inclusion criteria for the clinical study were as follows: (i) aldehyde dehydrogenase 2 (ALDH2) genotype: ALDH2*1/*1; (ii) healthy males, free of chronic illness such as liver, kidney, heart, gastrointestinal, lung, endocrine, metabolic diseases, and mental disorders; (iii) the ability to drink 1.2 g/kg alcohol as a Japanese distilled spirit (Shochu) and conduct abstinence according to the protocol. ALDH2*1/*2 or ALDH2*2/*2 subjects were excluded as they have a genetic intolerance to drinking higher quantities of alcohol [16].

### Test meals

Both the commercially available tomato paste and dl-alanine were kindly provided by Kagome Co., Ltd (Tokyo, Japan) and Ajinomoto Healthy Supply, Inc. (Tokyo, Japan) respectively. Tomato paste (2,000 g), dl-alanine (250 g) and tap water (2,750 g) were mixed well and the mixture was prepared as freeze-dried products that yielded a total of 880 g. The dry AFT product was stored in a desiccator attached to a vacuum aspirator at room temperature for use in Experiments 1 and 2. A commercially available table sugar was used as a control meal and shochu (20 % alcohol, v/v) was utilized as the alcohol in all experiments.

### Study design

**Fig. 1:**
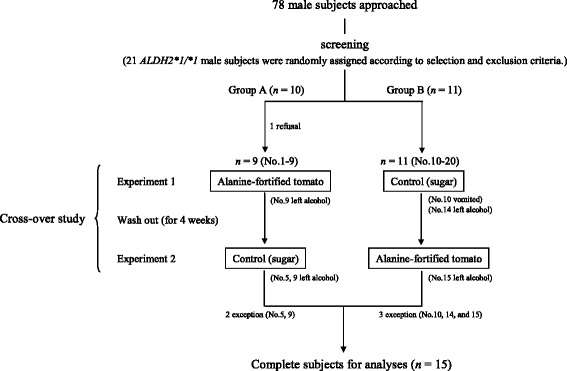
The study flowchart showing the randomized cross-over study that was performed during a 4-week period

**Fig. 2:**
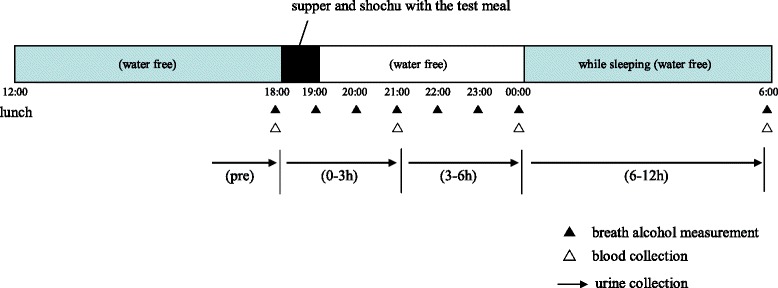
Timeline for experiment 1 and 2

A non-blind, single-center, randomized cross-over design was used for this study. The study flowchart is shown in Fig. 1. Subjects were scheduled to receive sugar (as a control) and AFT on two separate test days, 4 weeks apart in a randomized order. Prior to each Experiment (1 or 2), participants were instructed not to consume alcohol for two days prior. The timeline of the experiments (1 or 2) is shown in Fig. 2. On the day of the experiments, subjects were provided lunch at 12:00 pm in the Sekino Clinical Pharmacology Clinic (Tokyo, Japan). They had a free time until the start of the experiment at the institution. All of the subjects ingested a commercially available micronutrients-fortified food as meal (716 kcal, BALANCEUP, Asahi food & healthcare Co., Ltd., Japan), shochu (1.2 g alcohol/kg body weight), and the mixed dry product of AFT (40 g, 150 kcal). The AFT was equivalent to approximately 400 g of raw tomato with 10.4 g alanine or sugar (38 g, 152 kcal) used as the control. All subjects refrained from eating foods except for the supper provided and the test meals. However, they were allowed to freely consume water throughout the trial. The volume of any consumed water and the excreted urine were recorded during the experiment. The energy and nutrient contents of the supper and each test meal are shown in Table 1. The subjects ingested the supper and the alcoholic drink containing the test meal within 1 h. Peripheral blood specimens from the subjects were collected from the cubital vein at 18:00, 21:00, 00:00, and 6:00 (awakening time), as well as before and after the ingestion of each meal to determine the blood acetaldehyde, acetate, and plasma glucose levels. Serum alanine concentrations measured from each sample obtained at 18:00, 00:00, and 6:00. Breath alcohol concentrations (BrAC) were monitored using AlcoQuant® 6020 plus (EnviteC, Germany) as a breath alcohol analyzer at 18:00, 19:00, 20:00, 21:00, 22:00, 23:00, 00:00, and 6:00. Urine specimens were also collected from 12:00 to 18:00, 18:00 to 21:00, 21:00 to 00:00, and 00:00 to 6:00. The volume and pH was recorded for each of the four urine samples. The subjective sensations following alcohol consumption were evaluated based on the questionnaire data. The subjects were required to answer 14 questionnaires at the same time that the BrAC was determined. After a wash out period of 4 weeks, the cross-over trial was performed using the same protocol.

**Table 1:** Total energy and nutrition expenditure of the supper and the test meals

	Energy, kcal		716	
	Protein, g		16.4	
Supper	Fat, g		39.4	
	Carbonhydrate, g		74.0	
	Dietary fiber, g		12.0	
		Alanine-fotified tomato		Control (sugar)
	Energy, kcal	150		152
	Protein, g	14.1		0.0
Test meal		(alanine: 10.4 g)		
	Fat, g	0.6		0.0
	Carbonhydrate, g	18.2		38.0
	Dietary fiber, g	4.0		0.0
		(insoluble: 2.4 g)		

The amount of carbohydrate includes that of dietary fiber

### Determination of alcohol metabolites and biochemical assessment

The blood acetaldehyde concentration was measured using a 2, 4-dinitrophenylhydrazine derivative method for acetaldehyde by ultraviolet detection (365 nm) using a high-performance liquid chromatography (HPLC) technique used in a previous report [17]. The blood acetate level was determined using HPLC (Shimadzu organic acid determination system; Shimadzu, Japan) fitted with an ion-exclusion column and a conductivity detector [18]. Following collection, the blood samples (0.5 mL) were mixed with 1.0 mL of 0.5 N perchloric acid and the supernatants were prepared by centrifugation. The serum alanine concentration was analyzed using an automated precolumn derivatization amino acid analytical method based on HPLC/electrospray ionization mass spectrometry (UF-Amino Station system; Shimadzu, Japan) as previously described [19]. The levels of plasma glucose and vasopressin; blood vitamin B1; serum vitamin B6, folic acid and vitamin C; and urinary pH were measured by a local laboratory for clinical examination (SRL Inc., Tokyo, Japan).

### Subjective sensations

It is well established that alcohol produces both stimulant and sedative effects [20–22]. Martin et al reported the biphasic effects of alcohol on a scale (a seven-item stimulant subscale and a seven-item sedative subscale), which held promise as a self-reporting measure of the impact of alcohol [20]. The stimulant items included the descriptions of elated, energized, excited, stimulated, talkative, up, and vigorous. The sedative items included difficulty concentrating, down, heavy head, inactive, sedated, slow thoughts, and sluggish. Each subject reported on each the 14 items as a subjective sensation on a 10 cm visual analog scale (VAS). The distance (cm) from the left edge of the line to the mark placed by the subject was measured at each time point. This measurement was then used in subsequent analyses as the sensation score from 0 (not at all) to 10 (extremely) that best described their present feelings. The stimulant or sedative score added the score of each of the seven items, and the scores were expressed as the median, quartile as well as the lowest and highest data in box plots. Each of the seven items of the sedative subscale performed at 6:00 was box plotted in a similar way.

### Statistical analyses

All statistical analyses were performed using Dr. SPSS II software (SPSS Inc.). Each item between the AFT and control group was compared using a paired *t*-test. Temporal changes of each item were analyzed using a paired *t*-test or repeated ANOVA (if there were more than three points). These data were presented as mean ± SD. The acceptable level of significance was 5 and 1 % for each analysis. The VAS scores on subjective sensations between two conditions were evaluated using a Wilcoxon signed-rank test. A value of *p* < 0.05 was considered to be significant.

## Results

Of the 78 subjects recruited to the study, 21 subjects were randomly assigned to Group A or B. Of these, 20 subjects conducted the cross-over study in both Experiments 1 and 2 because one subject dropped out before the beginning of the cross-over study. Final analyses were performed using data from 15 subjects because four subjects left their alcoholic beverage, and one subject vomited after drinking the alcoholic beverage. The baseline characteristics at the time of screening for the 15 healthy male subjects who completed the study are shown in Table 2. Fifteen subjects consumed 73.6 ± 8.4 g of alcohol in the form of the distilled alcoholic beverage, shochu in each experiment. The volume of water that was consumed was 1,784 mL in the control group and 1,695 mL in the AFT group. The total urinary volumes were 1,569 mL in the control group and 1,561 mL in the AFT group. There were no significant differences observed in the both volumes between the control and AFT groups.

**Table 2:** Baseline characteristics of 15 healthy male subjects who completed the analyzes

Age, y	43.2 (15.8)
Weight, kg	61.4 (7.0)
Height, cm	170.7 (7.4)
BMI, kg/m2	21.0 (1.7)
Serum asparate aminotransferase, U/L	21 (4)
Serum alanine aminotransferase, U/L	19 (7)
Serum r-glutamyl transpeptidase, U/L	36 (23)
Serum lactate dehydrogenase, U/L	163 (19)
Serum creatine kinase, U/L	117 (53)
Serum creatinine, mg/dL	0.80 (0.08)
Serum urea nitrogen, mg/dL	12.4 (2.5)
Serum albmin, g/dL	4.7 (0.3)

Results are expressed as means and standard deviation (mean ± SD)

*BMI* body mass index

**Fig. 3:**
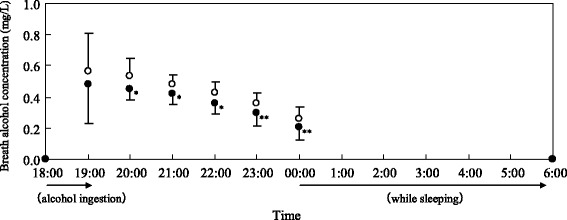
Concentration-time profiles of the breath alcohol levels of 15 healthy men after 1.2 g alcohol/kg body weight with a meal and AFT or sugar (as the control). Results are expressed as means and standard deviation. The control and AFT groups are represented by an open and closed circle, respectively. *p < 0.05, **p < 0.01 versus the control group. *AFT* alanine-fortified tomato

BrACs in the AFT group were significantly lower than those in the control group at 20:00, 21:00, 22:00, 23:00, and 00:00 after alcohol consumption (Fig. 3). However, there were no significant differences between both conditions at 19:00. The BrACs at 18:00 (before alcohol ingestion) and at 6:00 (awake time the next morning) were under the detection limit in all the subjects of both groups. The area under the curve that was calculated from the BrACs from 18:00 to of 00:00 was significantly lower for the AFT group (2.1 ± 0.5 mgL-1 × h) than for the control group (2.5 ± 0.5 mgL-1 × h). Blood acetaldehyde concentrations in both groups were similar after the alcohol challenge (Table 3). Acetate concentrations in the blood were significantly higher in the AFT group compared with the control group at 12:00 following alcohol consumption. There were no significant differences observed between the AFT and control groups at 18:00, 00:00, and 6:00. Plasma glucose levels in both groups exhibited no change over the course of the experiment, and the levels between the groups were not different at any time point. Serum alanine concentrations at 00:00 and 6:00 were significantly decreased compared with the values at 18:00 in the control group. The alanine levels at 00:00 and 6:00 were significantly elevated compared with the levels recorded at 18:00 in the AFT group. Furthermore, at the same time points, the alanine levels had significantly decreased in the control group when compared with those in the AFT group.

**Table 3:** Comparison of the levels of alcohol metabolites, nutrient, micronutrients, and physiological factors in the control or AFT groups

		18:00	21:00	00:00	6:00	Temporal change
		(Before alcohol ingestion)				
Alcohol metabolites						
Acetaldehyde (μg/mL)	Control	0 (0)	0.21 (0.20)	0.21 (0.22)	0.02 (0.04)	-
	AFT	0 (0)	0.33 (0.15)	0.16 (0.13)	0.02 (0.03)	-
Acetate (μg/mL)	Control	14.1 (3.8)	76.8 (20.3)	66.9 (15.1)	11.4 (3.5)	-
	AFT	12.4 (4.0)	97.8 (25.4)**	76.5 (15.2)	13.6 (3.8)	-
Nutrients, micronutrients						
Glucose (mg/dL)	Control	90 (4)	94 (14)	88 (8)	87 (8)	No significant
	AFT	91 (6)	86 (11)	89 (5)	88 (6)	No significant
Alanine (μmol/L)	Control	194 (57)	-	94 (30)	162 (45)	Significant decrease (*p* < 0.001)
	AFT	200 (44)	-	396 (122)**	290 (84)**	Significant increase (*p* < 0.001)
Vitamin B1 (ng/mL)	Control	5.9 (0.9)	-	-	6.4 (0.9)	Significant increase (*p* < 0.01)
	AFT	5.8 (1.0)	-	-	6.1 (1.0)	Significant increase (*p* < 0.001)
Vitamin B6 (ng/mL)	Control	12.0 (5.9)	-	-	14.8 (6.6)	Significant increase (*p* < 0.001)
	AFT	13.1 (5.6)	-	-	15.8 (6.1)	Significant increase (*p* < 0.001)
Folic acid (ng/mL)	Control	5.7 (1.9)	-	-	6.8 (2.1)	Significant increase (*p* < 0.01)
	AFT	5.5 (2.2)	-	-	7.7 (2.5)*	Significant increase (*p* < 0.001)
Vitamin C (μg/mL)	Control	7.0 (2.5)	-	-	6.4 (2.0)	Significant decrease (*p* < 0.05)
	AFT	7.2 (2.1)	-	-	7.0 (2.2)	No significant
Physiological factors						
Urinary pH	Control	6.7 (0.8)	5.7 (0.3)	5.8 (0.5)	5.7 (0.8)	Significant decrease (*p* < 0.05)
	AFT	6.7 (0.7)	6.1 (0.4)**	6.5 (0.5)**	5.8 (0.4)	Significant decrease (*p* < 0.001)
Vasopressin (pg/mL)	Control	4.3 (1.7)	4.5 (1.7)	5.5 (6.0)	6.3 (6.8)	No significant
	AFT	4.6 (1.8)	4.7 (2.1)	4.6 (1.6)	4.8 (4.8)	No significant

Results are expressed as the means and standard deviation (mean ± SD, *n* = 15). Asterisks indicate significant differences compared to the control group; *p < 0.05, **p < 0.01, Student’s *t*-test. Temporal changes of each item were analyzed with a paired *t*-test or a repeated ANOVA. -; not determined or analyzed. *AFT* alanine-fortified tomato

Blood vitamin B1, serum vitamin B6, and folic acid concentrations in both groups significantly increased at 6:00 compared with those at 18:00. The folic acid level in the AFT group was significantly higher than that in the control group at 6:00. The serum vitamin C level in the control group was significantly lower at 6:00 than at 18:00. In contrast, the vitamin C level in the AFT group exhibited no significant change over time. The urinary pH in both groups was significantly reduced after alcohol ingestion, with that in the AFT group at 21:00 and 00:00 being significantly higher than in the control group. Plasma vasopressin concentrations in both groups had no significant change.

**Fig. 4:**
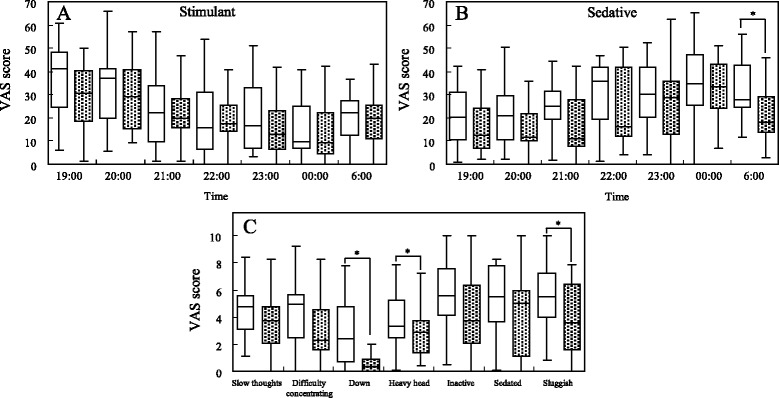
Box plots of the subjective scores for each drunkenness symptoms reported by the participants after the ingestion of control and AFT (median and quantiles, *n* = 15). The control and AFT groups are represented by an open and closed square, respectively **a**) Temporal changes of the sum of seven stimulant scores (elated, energized, excited, stimulated, talkative, up, and vigorous). **b** Temporal changes of the sum of the seven sedative scores (difficulty concentrating, down, heavy head, inactive, sedated, slow thoughts, and sluggish). **c** Temporal changes of each sedative score at 6:00. Asterisks indicate significant differences between the control and AFT groups; *p < 0.05, Wilcoxon signed-rank test. *AFT* alanine-fortified tomato

The stimulant and sedative scores produced following alcohol ingestion are presented as box plots in Fig. 4. The sedative score at 6:00 (the next morning) was significantly different between the AFT and control groups. The stimulant scores had no differences for any of the time points between both groups. Specifically, out of the seven items on the sedative subscale, the scores of the three items, down, heavy head, and sluggish exhibited significant differences between the AFT and control group. The three scores from the AFT group were significantly lower than those of the control group.

## Discussion

The purpose of this study was to confirm the relieving effect of AFT against alcohol-induced adverse effects. This effect is thought to be attributed to the ability of AFT to lower the alcohol concentration levels under the conditions of excess alcohol drinking with a meal. An alcohol dosage of 1.2–1.75 g/kg has been used in several studies that have examined the effects of food materials or constituents on intoxication or the presence of a hangover [23–27]. An alcohol dosage of 1.2 g/kg was used in this study because of the difficulty in evaluating the physiological effects of the subjects after drinking excessive alcohol. Higher dosage leads to an increase in the vomiting of test meals as a result of acute alcohol intoxication. Unfortunately, one subject (No.10) in the control group vomited during Experiment 1 and was exempted from analyses. Moreover, the 20 healthy subjects who participated in the cross-over study were asked whether they were experiencing hangover symptoms (yes or no) on the subjective feeling score the next morning in both experiments. In our results, six subjects experienced hangover symptoms (response: yes; 30.0 %) in the control group, compared to only one individual (5.0 %) in the AFT group.

Deficiencies in the vitamin B complex, folic acid, and vitamin C are well recognized in alcohol abusers or alcoholics [28–32]. Alcohol likely affects the absorption or metabolism of these vitamins. Thus, subjects in both experiments ingested the micronutrients-fortified foods as the provided meal to minimize the decrease in the blood vitamin levels from the excessive alcohol drinking. Therefore, the blood vitamin concentrations, apart from vitamin C (present in only small amounts in the foods consumed) significantly increased after alcohol ingestion. Blood vitamin C level in the AFT group did not decrease because tomatoes provided vitamin C to the subjects. Therefore, this study was conducted under the conditions of supplementation for the subjects via calories and micronutrients derived from the provided meal. In this regard, this study examined some variations related to acute ingestion of alcohol. Further studies are warranted to make sure of the effects of supplementation toward nutrients status in a body due to chronic alcohol ingestion.

In our results, we demonstrated that the combination of tomato and alanine decreased the BrAC in healthy male subjects. We believe that insoluble dietary fiber that exerts a synergistic action with alanine in tomatoes observed in previous studies [14, 15] is the main mechanism by which this effect occurs. In addition, we confirmed that the ethanol-maintaining ability of dietary fiber in delaying the gastric emptying in the alcohol absorption process vanished because of heat processing under the dried condition of that (data not shown). The 12 g of dietary fiber in the meal was relevant for determining the effect of deactivation by thermal sterilization.

Blood acetaldehyde levels between both groups were not different. The peak values of blood acetaldehyde after ingesting of 0.4 g/kg alcohol in healthy Japanese men are ALDH2*1/*1: 4.1 μmol/L, ALDH2*1/*2: 23.4 μmol/L, and ALDH2*2/*2: 79.3 μmol/L, respectively, as described by Mizoi et al. [33]. The peak acetaldehyde level of 0.21 or 0.33 μg/mL (4.7 or 7.5 μmol/L) found in ALDH2*1/*1 subjects after ingesting 1.2 g/kg alcohol in this study was lower than those of the ALDH2*1/*2 and *2/*2 types that received 0.4 g/kg of alcohol. Therefore, it seems that acetaldehyde is rapidly metabolized to acetate in both conditions, even following excessive alcohol consumption. Thus, the acetaldehyde levels would have had little effect on the physiological actions and subjective sensations that were obtained. However, blood acetate level in the AFT group was higher than that in the control group. It is suggested that an increased level of blood acetate during alcohol oxidation may be used as an indicator of enhanced alcohol elimination [34]. As reported by Smith et al., it is likely that if the absorption rate of the alcohol dose is sufficiently slow to maintain the portal alcohol level below the Vmax of hepatic alcohol dehydrogenase, some alcohol will escape into the peripheral circulation in low level [35]. Inhibition of gastric emptying would result in a decrease of the portal alcohol level. Thus, we believe that the combination of alanine and tomato effectively enhances alcohol elimination due to delayed gastric emptying in humans.

Some alterations of the metabolic functionality of the liver and other organs occurs in response to the presence of alcohol in the body and can result in low blood glucose [36], as well as low alanine levels [5]. The blood glucose levels only decreased a minor amount in both conditions, likely because of the glucide intake from the supper and test meals that provided glucose supplementation. In addition, the sensation score from the subjects after alcohol ingestion was undoubtedly not related to symptoms of hypoglycemia. The increase of alanine in the blood is likely to reflect a sufficient supply of NAD+, which is necessary to facilitate alcohol metabolism. Alanine supplementation that led to the elevated plasma alanine concentration in the AFT group was considered to be nutritionally essential for consecutive alcohol metabolism. Several hormonal alterations have been observed during the excessive ingestion of alcohol. In particular, hangover severity is likely proportional to the antidiuretic hormone level and metabolic acidosis [37].

Alcohol causes the body to increase urinary output by inhibiting the release of anti-diuretic hormone (vasopressin), which prevents the kidneys from reabsorbing water. However, vasopressin levels increase as alcohol levels decline to zero, and during a hangover, this condition can result in additional fluid loss [38]. In our study, the plasma vasopressin levels in both groups remained relatively unchanged throughout the experiments. The volume of water that was consumed was greater than the total urinary volume in both groups. These results implied that the subjects experienced little fluid loss (dehydration) the next morning.

Eggleton et al. reported that urinary pH in all subjects who consumed 40 g of alcohol were lower than that found in control group [39]. In this study, the urinary pH in both groups was significantly decreased. However, the pH of the AFT group was significantly higher than the control group at 21:00 and 00:00. In one study, the blood pH in a severe hangover group was markedly lower than that in a mild hangover group after 1.5 g/kg alcohol consumption and there was a good correlation between the hangover scale values and blood pH [40]. The decreased severity in urinary pH changes in the AFT group could be due to the lowering of blood alcohol levels during the experiment.

Self-reported (subjective) alcohol effects have often been measured with ratings of how intoxicated subjects perceive themselves. Alcohol produces both stimulant and sedative effects, in generally, the stimulant ratings are higher than sedative ratings during rising blood alcohol levels, and sedative ratings are higher than stimulant ratings during falling blood alcohol levels [21]. Our results indicated similar tendencies (see Fig. 4), as the stimulant effects in both conditions expressed as short-term effects in the initial phase of alcohol consumption, and the sedative effects confirmed as long-term ones in the control group from several hours later to the next morning. However, a sedative score of AFT condition at 6:00 (awakening time) was significantly lower than that of the control group. In particular, down, heavy head, and sluggish scores in the sedative items of AFT condition at 6:00 were significantly decreased than that of the control group. The morning after a night of excessive alcohol drinking, people wake up with unpleasant feelings. The experienced sensations are caused by the alcohol hangover that develops when alcohol disappears from the body. The hangover severity is most pronounced at 12 to 14 h after the starting of alcohol consumption [41]. Previous studies demonstrated that an alcohol dosage that produces a peak blood alcohol level of at least 0.11 to 0.12 % is necessary to develop an alcohol hangover [42]. In the present study, the estimated peak average blood alcohol concentrations in the subjects that were calculated from breath alcohol levels were 0.12 % in the control group and 0.10 % in the AFT group. The levels were close to that of being consciousness of a hangover. Thus, the highly retaining sedative scores resulted from discomfort sensations continued until next morning. Decreased sedative scores in AFT condition next morning could be considered because of the lowering alcohol levels during the experiment. There were no significant differences between the scores of the AFT group and those of the control group because of the existence of alcohol in the body during 19:00 to 00:00. This is despite the significant differences in the alcohol levels for both groups. Millar et al. [43] reported that subjective intoxication was equivalent in fed and fasted conditions for a high alcohol dose, although the meal ingestion prior to the consumption of alcohol reduced the peak blood alcohol level, which was similar to our results.

With regard to the limitations of this study, the randomized cross-over study conducted was an open-label design. We were not able to produce placebo foods that were confused with the alanine-fortified tomatoes. Thus, the significant difference between two groups did not allow adequate assessment of the effect of AFT ingestion. Accordingly, a double-blind trial needs to be performed using placebo foods to confirm our findings.

## Conclusion

The BrAC and urinary pH alterations due to alcohol-induced metabolic acidosis were significantly decreased by the ingestion of AFT. Several subjective sensations after alcohol ingestion were also significantly reduced by AFT the morning after alcohol consumption. This study demonstrated that the simultaneous ingestion of AFT with excess alcohol under the condition of micronutrient-fortified meal consumption reduced the acute alcohol-induced adverse effects. This effect of AFT was found to be a result of the decreased levels of alcohol in healthy human volunteers and may have the potential to be used in a therapeutic or precautionary context in the future.

## Abbreviations

AFT: alanine-fortified tomato
ALDH: aldehyde dehydrogenase
BAC: blood alcohol concentration
BrAC: Breath alcohol concentration
NAD+: nicotine adenine dinucleotide
